# The Transactions of the Provincial Medical and Surgical Association

**Published:** 1844-01

**Authors:** 


### Art. IV.

*The Transactions of the Provincial Medical and Surgical Association.* Vol. XI.—*London*, 1843. 8vo, pp. 568.

This volume is more than usually scanty in practical communications, which may be owing in part to the weekly journal of the association affording a more ready channel in publication, and not, we trust, to any greater apathy of the provincial practitioners to promulgate in a durable form some of the results of their large experience. We well know the exertion required, (particularly to one but little accustomed to composition, and whose time is very fully occupied in the distracting duties of an active medical practice,) to sit down and to write for the press; but is it not a duty which elder practitioners owe, both to society and to their own profession, not to let their experience die with them, but to communicate modes of treatment, which, by long experience, they have found to be beneficial, and of the truth of which they are sure, to their younger and less experienced brethren?

There are enough and to spare of systematic books and treatises, in which an attempt is made to treat fully and completely a science or parts of it which is incomplete and fragmentary. What is wanted is, that those who are experienced should communicate as briefly as they choose what they know about the treatment of those diseases on which they have fully made up their minds, stating those rules in distinguishing diseases, or in judging their progress or termination by which they themselves are guided, and giving hints as to remedies, sick room management, and similar details apparently small but really most important, such as they themselves would have been glad of in their earlier career, and which are now so willingly read and used by those who go to books in their difficulties—not a small number in this reading age. One of the chief benefits of the Provincial Association was to afford a stimulus to such a useful exertion, and a channel for the results; and we regret that this volume should not contain more proofs that this object has been more fully accomplished. Not more than sixty pages out of 567 are occupied with original papers on practical subjects. These few we shall analyse, with the exception of the essay of Mr. Turner, On Dislocations of the Astragalus, which we reserve for a separate notice on another occasion.

Art. iii. *Experimental and Practical Researches on the structure and function of Blood-corpuscles, on Inflammation, and on the Origin and Nature of Tubercles of the Lungs*, by William Addison, f.l.s. This is a paper of great interest, containing, as it does, the results of a very extended series of microscopic inquiries, on which the author has been for some time engaged. Some of these results, to which no doubt can be reasonably attached, are of great value; others, we fear, will not stand the test of further investigation, particularly with a microscope of qualities superior to that which we understand Mr. Addison to have been in the habit of employing. Knowing what differences of opinion are liable to occur even among those possessed of the best instruments, we cannot be satisfied with conclusions based upon researches carried on with one of inferior quality. We shall briefly indicate, for the guidance of our readers, those points in Mr. Addison's researches which we deem most worthy of favorable attention, and those which must, in our opinion, be received with caution.

In the first section, on the *blood-corpuscles of man*, the author directs attention prominently to the *colourless* corpuscles, which, on account of their correspondence in size and outline to the red discs, are very apt to escape notice. There can be no reasonable doubt that these are identical in character with the (so called) lymph-globules of the lower vertebrata, which are readily distinguished from *their* red discs by the large size of the latter; they are acted upon in a peculiar manner by liquor potassæ, being made to burst and discharge a number of contained granules. Mr. Addison directs attention to the fibrous arrangement which fibrin presents in coagulating, and which may be well seen in watching the coagulation of a small quantity of the upper stratum of sizy blood under the microscope. To this circumstance Mr. Gulliver also has attracted notice, and there seems much reason to believe that the simple coagulation of fibrin in this form is the origin of the ordinary fibrous tissues, of which Dr. Carpenter has pointed out a beautiful example in the membranous basis of the egg-shell.

In section ii. on pus corpuscles, the author expresses the opinion—founded on their correspondence in appearance and in behaviour with liquor potassæ, with the colourless corpuscles of the blood—that the two have the same origin, and concludes "that pus corpuscles of all kinds are altered, colourless blood-corpuscles; and that all limpid, colourless, abnormal discharges, are effusions of the liquor sanguinis, containing more or less of the fibrin and corpuscles in varying proportions." In regard to this conclusion, though it accords with that of Dr. Barry, we must take leave to express some doubt. It seems to us that the pus corpuscles have something too specific in their *character*, if not in their *appearance*, to enable us to regard them as merely altered colourless corpuscles; and we think the idea of Dr. Carpenter a probable one, that they hold the same relation to the *liquor puris*, as the colourless corpuscles to the *liquor sanguinis*. (Report on Cells, § 48.)

In the third section, on the blood-corpuscles and on the lymph-globules of the frog, Mr. Addison endeavours to prove that the latter constitute "the inner vesicle or central portion of the red corpuscle." This we think an untenable position, for reasons which we cannot now enter upon fully; but the marked want of correspondence between the sizes of the two classes of corpuscles in vertebrata, and the total absence of the red discs in invertebrata, seems to us a sufficient objection to this view, until much more satisfactory evidence be adduced in its favour, than that brought forward by Mr. Addison.

The fourth section, on inflammation, contains little else than an account of the changes in the number and movements of the lymph-corpuscles of frog's blood, which were witnessed by Mr. Addison, as a consequence of the application of irritants. This subject is one of extreme interest, as opening an entirely new line of inquiry on this previously-exhausted subject; and, short as this portion of Mr. Addison's paper is, we consider it the most valuable of the whole. The inquiry cannot, however, be regarded as complete until it has been prosecuted in regard to warm as well as cold-blooded animals; since, as Dr. Macartney and others have pointed out, it is doubtful how far real inflammation can be said to take place in the latter. It will, perhaps, be more correct to say, that the colourless corpuscles accumulate and multiply in vessels actively engaged in the *formative process ;* a conclusion which harmonizes with Dr. Barry's results, and does not run counter to the existence of a similar condition in inflammation, in which there is a morbid exaggeration of that process.

The succeeding section, on cells, contains many interesting views of the functions and development of these constituents of the organism, whose importance in the animal as well as the vegetable economy is now coming to be generally acknowledged. Mr. Addison may fairly claim to be among the foremost in developing these views; but we cannot admit as proved, or even as probable, that the circulating cells (or in other words, the colourless corpuscles of the blood) ever became fixed, and transformed into the elements of tissue. Both they and the tissue-cells are descendents of the primordial cells of the embryo; but when once the separation between the stable *solids* and the circulating *fluids* is established, and their respective functions are specialized, we doubt the conversion of the cells of the latter into those of the former. The case of the epithelial cells, which both Mr. Addison and Mr. Barry consider the strongest in support of their view, appears to us the one most easily upset; since the researches of Mr. Bowman and Mr. Goodsir have shown the existence of a sheet of structureless membrane, lying beneath the epithelium and epidermis, and effectually separating them from the blood-vessels of the mucous membrane and skin, except so far as the transmission of *fluids* is concerned. This may be well seen in the ultimate tubuli and vesicles of the glands, which are formed of nothing but this basement or primary membrane, on the *outside* of which the vessels are distributed, whilst the epithelium cells line their interior. There is a continual re-formation of the latter, as a part of the process of secretion; and it seems to us beyond all probability that colourless blood-corpuscles should be continually escaping from the capillary vessels, passing through the basement membrane, and becoming transformed into epithelium cells on the other side. It seems to us that both the observers who uphold this doctrine, have been led into error by the resemblance which exists among nearly all cells at an early stage of their development.

An account of Mr. Addison's views on the aeriferous structure of the lungs, which he considers necessary to explain the nature and primary seat of tubercles, constitutes the sixth section. These views have been communicated to the Royal Society, and published in the Philosophical Transactions. In opposition to the doctrine of Reissesien and others, Mr. Addison thinks he has derived ample evidence from various experiments "that the bronchial tubes, after subdividing into a multitude of minute ramifications, which take their course in the cellular insterstices of the lobules, terminate in their interior in symmetrically branched air-passages and freely-communicating cells." He considers these to be formed at the first inspiration by the pushing forward and distension of the membrane composing the ultimate bronchial subdivisions. "The symmetrically-branched air-passages, thus formed by respiration, are no longer tubes. I have, therefore, distinguished them by the term 'lobular passages;' and a section of these passages shows the *oval foramina*, leading from cell to cell, so conspicuous in a thin layer of inflated and dried lung. The air-cells of the lungs, then, are formed at birth, by the pressure of the atmosphere acting upon the extremities and against the sides of the *intralobular* bronchial subdivisions. They have not a general and indiscriminate inter-communication, for there are no anastomoses between the intralobular subdivisions of the bronchi; therefore, the cells forming one lobular passage have no communication with those of the adjoining ones, except by their common opening into a larger ramification." These views differ from those recently promulgated by M. Bourgery, chiefly in this,—that the latter speaks of the lobular passages (under the designation of *canaux labyrinthiques aërifères*) as turning back at the boundary of a lobule to reenter its interior, and terminate in some of the deeper canals. As to the general fact that the bronchial tube terminates in a series of cells, communicating with each other by oval foramina, we think that the inquiries of Mr. Addison and M. Bourgery leave no doubt. As to the mode of their production, however, we incline rather to the opinion of the latter, who seems to consider that these cells are formed like other cells, and that the air finds its way into them by the rupture of their walls at a particular spot, so that a linear series of cells becomes converted into a passage; and who describes this process as taking place during the whole period of youth, so that the number of air-cells in the lungs continues to increase up to adult age, after which the number diminishes. The idea of the formation of the air-cells by the act of inspiration, entertained by Mr. Addison, seems to us to be too mechanical. "The blood-vessels," continues Mr. Addison, "lie exterior to or between the lobular passages; and as the membrane forming one of these passages is pressed by the inspired air into close contact with that of the adjoining ones, it follows, that the capillary blood-channels ramify or run between two membranous layers, and any increase in the diameter of these channels must separate these layers."

Mr. Addison's memoir concludes with an account of his views on the structure and character of tubercle. "If a tubercle, or even the tissue of the lung near it, be slightly compressed between two slips of glass with a drop of water, it will crumble down and break to pieces; the fluid at the same time being rendered quite white or milky. This white appearance is attributable to a great number of minute objects, the assemblage of which constitutes the substance of the tubercle. They consist, for the most part, of molecules, granules, and granulated corpuscles, of various sizes; of aggregated granules without any tunic; and of collapsed tunics without any granules. These objects are mingled with a great many shapeless flakes and filaments, which are no doubt fragments of the membrane of the air-cells and of the minute blood-vessels, which, when involved in a tubercle, become so extremely brittle, that they must necessarily form a considerable proportion of the objects occupying the field of the microscope. The granulated corpuscles of a tubercle are sometimes very large, and the molecules and granules, which are very conspicuous, may frequently be seen upon the point of escaping from them." The similarity of these corpuscles to the colourless corpuscles of blood, leads Mr. Addison to the conclusion of their identity, to which we think the same objection applies, as to his similar idea in regard to the pus-globules and the epithelium cells. "The semi-transparent forms of tubercle and tubercular infiltrations owe their peculiarity to a great relative amount of granulated corpuscles; whereas the opaque white forms of tubercle are attributable to great numbers of isolated granules. Tubercles of the lungs are extremely common. They are at first visible as minute white-rounded bodies, dispersed at more or less distant intervals in the vesicular tissue of these organs; and very frequently they elude observation, not being discernible unless specially searched for with a lens, in thin macerated sections of the lung, slightly extended on a dark surface." Mr. Addison considers that "there is no distinction whatever between the spots of lepra in the skin, and tubercles in the lungs; if we except those arising from the different situation and function of the two tissues." The essential character of both diseases he believes to be an abnormal accumulation of epithelium cells, which produces more injury in the lungs than on the skin, on account of the delicacy and vascularity, and functional importance of the former; and also because the accumulation cannot be readily discharged. And, according to the view already stated, these cells he regards as neither more nor less than altered colourless corpuscles of the blood. "If the matter deposited in the air-cells in cases of pneumonia, and termed 'hepatization,' be examined by the microscope, objects in all respects similar to those which compose a tubercle are seen, mingled with pus-corpuscles." We believe that the view of Gerber and Gulliver will be found to be more correct—that the matter thrown out in pneumonia occurring in a healthy subject, is *organizable liquor sanguinis*; whilst the essential character of a tubercular deposit is *unorganizability*, indicated by its granular character, and the imperfect formation of its cells. Between the two extremes, there seem (as might be expected) to be all grades of intermediate conditions, perfect cells and fibres presenting themselves, to a greater or less extent, in deposits which are ranked as tubercular. For some remarks upon the observations of Gerber and Gulliver, which set this matter in what we conceive to be its true light, we may refer to Dr. Carpenter's Human Physiology, §§ 609 and 715.

Art. iv. *On the Long Issue in the Scalp*, by Dr. Wallis. Dr. Pritchard some time since directed notice to the advantages of a free incision through the scalp in chronic cerebral diseases, and from this paper it appears to be a practice much used in the Bristol Infirmary; Dr. Wallis who is one of the Physicians of that institution, having been led to adopt it from the surgical practice of Mr. Smith, the senior surgeon, who in cases of severe injury of the head without symptom of fracture or depression found a free incision through the scalp useful both from the local loss of blood, and the wound becoming an advantageous counter-irritant.

The incision is made in the following manner:

"Let the head be shaved entirely, and have the patient brought near to the right side of the bed; raise the head by a hard pillow, and put a towel round his neck to receive the blood; let an assistant keep the head steady, and at the same time draw the scalp downwards in all directions, so as to strain the calvarium as much as possible; the scalp will divide with so much more ease. In this, your own left hand will materially assist, by placing it at the upper and back part of the head, commencing the incision between your thumb and fore-finger, as far back as the lambdoidal suture; press the scalpel sufficiently down so as to divide the scalp entirely through at once; carry on the incision directly along the sagittal suture as far as the hair grows on the scalp, and which will cover the cicatrix after the issue is healed up. The length of the incision thus made will be in the adult about seven or eight inches; take care that the scalp be divided entirely, and perfectly through, so that the edges of incision will separate so far as to enable you to introduce a dossil of lint, rolled up hard, as thick as two fingers, and which should be well soaked in spirit of turpentine; this answers the double purpose of increasing the effect of the incision, and makes suppuration come on earlier, and will usually assist in stopping a further loss of blood. There is seldom more than six or seven ounces of blood lost. In those cases where depletion has been carried to a sufficient extent, and the further loss of blood is unadvisable, it may be prevented in the following manner: The instant the incision is completed, close the sides of the wound and make pressure upon it with your hand, whilst your assistant hands the lint, well soaked in the spirit of turpentine, and rolled up firmly of a proper length, so as not to extend beyond the extreme length of the incision, as it would be inconvenient in strapping down the wound sufficiently to check the flow of blood; a little flour and dry lint may be superadded, but the dossil must not be made so thick as to rise much above the edges of the wound, or else the adhesive straps will not be secure by being elevated, and thereby prevented from adhering near the edges of the incision. Should the incision be imperfectly made, that is to say, not entirely through the scalp, the arteries may only be partially divided; in which case they will continue to bleed, notwithstanding the pressure you may have made; of course the arteries will require to be completely divided, and allow them to retract and cease to bleed."

When there is much restlessness and delirium, and a risk that the dressings may be disturbed, and a further loss of blood is not desirable, Dr. Wallis applies the actual cautery to the bleeding vessels by touching them with the common thick plaster-knife heated in the fire. The adhesive straps should be an inch wide, and ten inches long, supported by a double-headed bandage, removed the next day, and replaced by a bread-and-water poultice on the top of the lint. When the lint comes out, another similar dossil may be introduced, and in a few days a double row of peas, seventy or eighty strung together, filling up the issue, which is to be kept open three or four months. Most cases also require caustic to be applied every five or six days, to ensure the due discharges. Three cases are detailed of amaurosis with pains in the head, giddiness, and other symptoms of chronic cerebral disease. In two of these the incision was followed by restoration of sight, and removal of the head symptoms, in the third by some improvement.

Nine cases follow of hemiplegia from cerebral apoplexy. All were bad cases, the symptoms either unmitigated by ordinary depletion, or increasing in severity. In the majority, the immediate improvement from the incision was marked, and the return either to perfect restoration of power or to mere improvement, was gradual.

Dr. Wallis states that this remedy is useful in epilepsy, depending on chronic diseases of the brain; one case is given in which it was of marked benefit, but the age of the patient is not stated, nor had a sufficient time elapsed after the operation to decide as to its curative effect.

In hydrocephalus Dr. Wallis asserts that the incision, if made before effusion has taken place, may prevent its occurrence. But no cases are given to prove this. Two cases are given of convulsions and insensibility following scarlatina in which the incision was followed by a return of consciousness and a cure; but a third case is also reported of a brother of one of the patients in which the same symptoms were relieved by a blister down the spine. We need hardly point out the difference between these cases and those of genuine hydrocephalus; the symptoms in both may depend on serous effusion, but in the latter disease a slow inflammatory condition of the brain has preceded the effusion, whereas in the former an effusion of serum seems often to take place in the brain from a general dropsical disposition of the body, and from which patients often recover with great rapidity. In genuine hydrocephalus, such a stage is, as far as we know, past hope.

Two cases of genuine hydrocephalus are reported in which the incision was made after the symptoms of effusion had set in; there was some improvement after it, but both patients died.

Three cases called inflammation of the membranes of the brain, (symptoms not given,) in which vigorous depletion had been used without relief, and the incision was followed by conspicuous improvement and recovery. Dr. Wallis states that these are but a few of the cases of the same disease which have happened in his practice, and that "no one case was unsuccessful where the incision was made before a destructive effusion had occurred."

In one case of delirium tremens in which depletion had been tried without benefit, and opium increased the restlessness, the incision removed the symptoms, but the patient returned to his old habits of drinking, and died suddenly.

Four cases of fever are reported attended in the early stages with irritation of the gastro-intestinal mucous membrane, and in the latter with delirium, insensibility, involuntary evacuations indicating effusion in the brain, and a case of scarlatina with the same cerebral symptoms, in all of which the incision was made, and the patients improved and recovered. In a sixth, the relief was temporary, and in the seventh the issue was fatal from loss of blood, the patient having torn off the dressings.

In erysipelas of the face and head with cerebral symptoms, Dr. Wallis thinks the incision very valuable as it relieves both conditions, the disease of the skin and the brain. Two cases are given.

The paper concludes with a case of hysteria in which the fit of unconsciousness lasted four days. The incision was made, the patient awoke, rallied, and we can easily believe Dr. Wallis when he adds, "I never saw her in the house afterwards."

This very severe remedy, an incision through the scalp to the bone seven inches long, and converted into an issue capable of holding eighty peas, is one, which, it is needless to say, should not be adopted unless other means fail. But in diseases of the brain of a very hopeless character, when ordinary means do not relieve, then sometimes, perhaps, "melius est anceps remedium quam nullum;" but the Lord save us from Dr. Wallis and his issue!

Art. v. *Case of Fracture of the Spine, treated successfully by extension;* by W. H. Crowfoot, of Beccles. A. C., æt. forty-two, whilst driving a carriage under an archway was bent double by the back of his neck coming in contact with a beam. Complete paralysis both of sensation and motion of both legs, great deformity about the ninth to the twelfth dorsal vertebra, with posterior curvature, the spinous processes of the ninth and tenth vertebræ were divided from each other, the body of the ninth having been forced forward, whilst that of the tenth projected backwards: inability to empty the bladder. No doubt existed of displacement of the bones of the spine and pressure on the cord. A broad and well-padded belt was passed round the chest and under the arms, and it was fixed from behind to a strong staple at the upper end of the frame of the bed: a similar belt was buckled round the body just above the pelvis with two strong straps attached to it, one before and the other behind, each having a strong iron ring at its extremity; these straps were brought between the thighs and made fast to the pulleys. Gradual but considerable extension was now made which evidently diminished the curvature and restored a certain degree of sensibility to the legs. This excited a hope that if the bones could be retained in their amended position and means were taken to obviate inflammation the cord might resume its functions. The patient was placed on his back on a firm bed, so that his fæces could be removed without disturbance and the most perfect rest enjoined. In the first ten days, from six to ten ounces of blood were daily taken away from each side of the spine alternately, with great relief to the patient's feelings, who was cautiously moved on his side by the sheet. The patient steadily improved; in three weeks he could move the right toe; in two months he could support himself with but little assistance, and in twelve months was able to resume his work as a coachman. There remains some deformity and an unusual separation between the ninth and tenth vertebræ, and horse exercise, or even walking, is apt to produce pain and numbness, otherwise he is in perfect health.

We give this case, although a single one, from its importance and rarity. The treatment is highly creditable to Mr. Crowfoot, and the whole paper (short though it be) indicative of a man of excellent sense and judgment.

ART. VI. *Case of Paralysis of the Serratus Magnus, which caused the lower angles of the right and left Scapulæ to become disengaged from the Latissimus Dorsi.* By JOHN M. BANNER, Esq. The above title and the following extract give the main features of this somewhat curious case.

"The circumstances which more particularly strikes the attention in the present case is the appearance of the scapulæ, more especially when the man attempts to raise and make use of his arms. In the quiescent position, the base of the bone, instead of lying parallel to the spine, is approximated to it at the lower angle, and stands out from the ribs a distance of two inches, leaving between the scapulæ a deep hollow; the upper angle being drawn high up in the neck, appearing on both sides, to the observer in front, midway between the shoulder and ear. The clavicle is in its natural position, as is also the acromion process of the scapulæ, to which it is articulated. When the patient attempts to raise the arm all these appearances are much increased. The base of the scapula approaches nearly to a right angle with the spine, forming, with the base of the scapula of the opposite side, a very obtuse angle, and both stand out about three inches from the ribs; the arm cannot be raised beyond the horizontal position. After stooping to a right angle to the lower extremities he is quite unable to recover the erect position without help, and there is an evident lateral curvature of the spine; both which may be consequences of the power of the longissimus dorsi, the sacro-lumbales, and the deep-seated extensors of the spine. In his ordinary position the upper part of the body is thrown back, evidently to balance the weight of the head and part of the body, which in the healthy person is supported in the completely erect position by the before named muscles." (pp. 344-5.)

ART. VII. *On Matico;* by Dr. JEFFREYS, of Liverpool. This paper contains a botanical description of the Piper angustifolium or Matico, and some cases in which it was used successfully as a styptic. The application of the under side of the leaf to leech bites which bleed profusely, and to small arteries which have been wounded, seems decidedly to arrest the hemorrhage; taken internally as an infusion it has been useful in hemorrhage from the intestines and in discharges from the urethra and vagina. Dr. Jeffreys has brought forward sufficient evidence of its value as a styptic to induce a fair trial to be given to it. There are two kinds of matico imported from South America, green and yellow; the latter is the ripe plant and should be obtained. The infusion is made with half an ounce to a pint of water, and the dose is three tablespoonsful four times daily.

---

